# Mortality in septic patients treated with short-acting betablockers: a comprehensive meta-analysis of randomized controlled trials

**DOI:** 10.1186/s13054-024-05174-w

**Published:** 2024-11-27

**Authors:** Mihai-Gabriel Alexandru, Patrick Niewald, Stefan Krüger, Rainer Borgstedt, Tony Whitehouse, Mervyn Singer, Sebastian Rehberg, Sean S. Scholz

**Affiliations:** 1 Department of Anaesthesiology, Intensive Care, Emergency Medicine, Transfusion Medicine and Pain Therapy, University Hospital of Bielefeld, Campus Bielefeld-Bethel, University of Bielefeld, Burgsteig 13, Haus Gilead I, 33617 Bielefeld, Germany; 2 Department of Anaesthesia and Intensive Care, Queen Elizabeth Hospital Birmingham, Birmingham, UK; 3 Bloomsbury Institute of Intensive Care Medicine, University College London, London, UK

**Keywords:** Betablocker, Landiolol, Esmolol, Septic shock, Mortality, Sepsis

## Abstract

**Background:**

Treatment with short-acting betablockers in septic patients remains controversial. Two recent large multicenter trials have provided additional evidence on this therapeutic approach. We thus performed a meta-analysis, including the most recent data, to evaluate the potential impacts of treatment with short-acting betablockers on mortality in adult septic patients.

**Methods:**

The data search included PubMed, Web of Science, ClinicalTrials.gov and the Cochrane Library. A meta-analysis of all eligible peer-reviewed studies was performed in accordance with the PRISMA statement. Only randomized, controlled studies with valid classifications of sepsis and intravenous treatment with short-acting betablockers (landiolol or esmolol) were included. Short-term mortality served as the primary endpoint. Secondary endpoints included effects on short-term mortality regarding patient age and cardiac rhythm.

**Results:**

A total of seven studies summarizing 854 patients fulfilled the predefined criteria and were included. Short-term mortality as well as pooled mortality (longest period of data on mortality) was not significantly impacted by treatment with short-acting betablockers when compared to the reference treatment (Risk difference, − 0.10 [95% CI, − 0.22 to 0.02]; *p* = 0.11; *p* for Cochran’s Q test = 0.001; I^2^ = 73%). No difference was seen when comparing patients aged < 65 versus ≥ 65 years (*p* = 0.11) or sinus tachycardia with atrial fibrillation (*p* = 0.27). Despite statistical heterogeneity, no significant publication bias was observed.

**Conclusion:**

Administration of short-acting betablockers did not reduce short-term mortality in septic patients with persistent tachycardia. Future studies should also provide extensive hemodynamic data to enable characterization of cardiac function before and during treatment.

**Supplementary Information:**

The online version contains supplementary material available at 10.1186/s13054-024-05174-w.

## Background

Sepsis represents a life-threatening condition estimated to affect > 48 million patients per year worldwide, with > 10 million deaths representing nearly 20% of all global deaths [1]. The United States and Europe are particularly impacted due to disproportionately high costs and increasing numbers of cases [2–5]. Various treatment strategies have been trialled in critically ill patients, including supportive therapy using short-acting betablockers to counteract excessive sympathetic activation [6–8]. Excessive catecholamine levels are associated with the severity of critical illness, complications, and high mortality rates [8, 9]. Achieving acceptable heart rates in euvolemic tachycardic patients is used as a readily available surrogate of sympathetic control [9–11]. Betablocker treatment may also be associated with hemodynamic advantages such as increased stroke volume and even increased mean arterial pressure [11]. Potential negative effects of beta_1_-blockade in sepsis include risks of arterial hypotension and bradycardia specifically under hypovolemic conditions and in patients with reduced cardiac contractility. Short-acting beta-blocking agents with high beta_1_ selectivity such as esmolol or landiolol were deemed to be ideally suited for this purpose in patients with sepsis, however heart rate control and mortality rates have only been evaluated in a limited number of studies [11–13].

In the light of four randomized controlled trials published within the last three years [14–17], diverging results [11–22], as well as limited quality of included trials [18–21], limited available evidence in previews systematic reviews [23, 24] and deviating inclusion criteria of previous meta-analyses [25, 26], we aimed to comprehensively analyze the available high quality research. This is mandatory to overcome potential uncertainties within the research and intensive care community regarding the use of betablockers in septic patients. As a result, we performed the present meta-analysis of randomized controlled trials and focused on mortality representing an unambiguous endpoint.

## Methods

This systematic review and meta-analysis was based on a pre-defined protocol, registered at the international PROSPERO database for prospective systematic reviews (CRD42023402150) and carried out in accordance with PRISMA Guidelines [27].

### Study protocol

A systematic literature search was completed for all peer-reviewed and published randomized controlled studies reporting the effects of short-acting betablockers (landiolol/ esmolol), when compared to standard care or placebo treatment. The patient population consisted of adult patients (aged ≥ 18 years) with sepsis, either defined by the Sepsis-3 criteria [13–16], or described as meeting two or more SIRS criteria (systemic inflammatory response syndrome) plus infection [12, 17], or described as presenting with septic shock requiring norepinephrine [11]. Studies were excluded if they could not provide valid data on mortality rates and on the timing of mortality assessment. There were no restrictions regarding the number of included patients. Of these, LANDI-SEP and STRESS-L explored the treatment effects on both a larger scale (LANDI-SEP, n = 196; STRESS-L, n = 126) and in a multicenter, prospective, randomized manner [28, 29]. Both studies used landiolol and focused on heart rate control, safety and efficacy as the primary endpoints. Mortality was evaluated as a secondary outcome [28, 29]. Short-term mortality was defined as 28-day mortality, or hospital mortality if 28-day mortality was not available [17]. Secondary analyses included biological heterogeneity (age < 65 versus ≥ 65 years) and the cardiac rhythm at treatment commencement. We were able to obtain individual patient data from recent trials [13–15] and compared these populations based on the type of cardiac rhythm at randomization, i.e. sinus tachycardia versus atrial fibrillation. Further analyses were performed regarding pooled mortality (longest period for data on mortality) and 90-day mortality as well as hospital mortality.

### Literature research and data extraction

Two investigators (M.A./P.N.) searched PubMed, Web of Science, ClinicalTrials.gov, and the Cochrane Library independently for eligible studies published until 31th August 2024. The search was performed using the terms: (short-acting beta-block* OR short-acting β-block* OR Ultrashort-acting beta-block OR Ultrashort-acting β-block* OR landiolol OR esmolol OR rapibloc OR brevibloc) AND (sepsis* OR septic OR critic*). Web of Science was searched using topic and articles, while PubMed was searched without restrictions. We also searched already published systematic reviews and meta-analyses and screened four additional studies and references [23–26]. Individual patient data on cardiac rhythm at the time of randomization were obtained through correspondence with the authors [14, 15] or from a subsequent analysis [30]. The same investigators screened the search results according to the title and abstract, reviewed the full text articles, considered whether the study was appropriate for inclusion, and extracted appropriate data from the publications [11–22, 30, 31].

### Assessment of bias

Quality of the included studies was assessed based on the risk of bias tool provided by Review Manager (RevMan) version 5.4.1. In case of disagreement between the two investigators, a third investigator (S.S.S.) was consulted.

### Statistical analysis

The effects of the intervention on mortality were investigated by assessing the risk difference between the betablocker and control groups by pooling available data on short-term, 90-day and hospital mortality. Subgroup analyses were performed with regard to potential heterogeneity. Hence, mean patient age was identified as potential confounder and included in sensitivity analysis. Further subgroup analysis was performed comparing atrial fibrillation with sinus tachycardia. Risk differences and pooled risk differences were determined and presented using Forest plots with respective 95% confidence intervals. A random-effects model (Mantel–Haenszel) was used to pool the data and estimate the results due to the presence of relevant statistical heterogeneity. Statistical heterogeneity between the trials was evaluated using Cochran’s Q Test and the I^2^ statistic as a measure of variability. The presence of relevant statistical heterogeneity was determined based on the outcomes of the Cochran’s Q Test, with a *p*-value < 0.05 and an I^2^ value > 50%. Potential publication bias was explored visually using Funnel plots. Asymmetry in the Funnel plot was considered as the presence of potential publication bias. Values were expressed as mean ± standard deviation (SD). Statistical analyses were performed using Review Manager (RevMan) version 5.4.1 (2014, Nordic Cochrane Centre, Cochrane Collaboration, Copenhagen, Denmark). A two-sided *p*-value ≤ 0.05 was considered statistically significant.

## Results

**Fig. 1:**
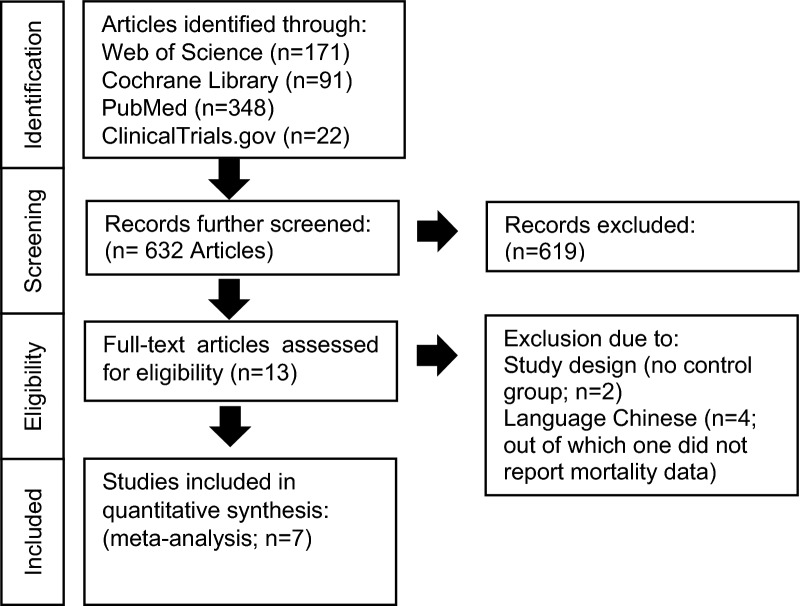
PRISMA flow diagram showing search and selection strategies

A total of 632 studies were identified through the initial literature search. Of these, 13 articles were identified as potentially appropriate after screening (Fig. 1). Following full-text review six studies were excluded due to study design (before-after; n = 2; [22, 31] and deviating study language (n = 4; published in Chinese) [18–21]. The seven trials that were included comprised a total of 854 patients and were randomized, unblinded, prospective, single- or multicenter studies (Table 1, Supplement Table 1). Inclusion criteria were age ≥ 18 years and sepsis (based on eligible classifications), tachycardia > 95[11, 12, 14, 15, 17] or > 100[13, 16] beats per minute (bpm), and need for vasopressor therapy to maintain MAP (mean arterial pressure) > 65 mmHg (Supplement Table 1). Exclusion criteria included pre-existing heart failure, severe atrioventricular disorders, limitation of therapy (do-not-resuscitate or intubate orders), and contraindications to receive the study drugs.

**Table 1:** Patient characteristics

Author (year)	Study design	Intervention	Comparator	28 days mortality (Betablocker/ Control)	Longest period for data on mortality (Betablocker / Control)
Rehberg (2024)	Multicenter, randomized controlled, open label	Landiolol; first 24 h after treatment start Titration to obtain HR of 80–94 bpm. Treatment for as long as vasopressors are mandatory; n = 98 (63 ♂/35 ♀)	Standard treatment; n = 98 (55 ♂/43 ♀)	43(98) / 39(97)	28 d: 43(98) / 39(97)
Whitehouse (2023)	Multicenter, randomized controlled, open label	Landiolol; After vasopressor therapy was needed for at least 24 h and after adequate fluid resuscitation. Titration to reach the target heart rate of 80–94 bpm. Treatment for up to 14 days after randomization or up to 12 h after vasopressor agents have been stopped. n = 62/63 (37♂/26♀)	Control group/Standard treatment: 63 (37♂/26♀)	23(62) / 16(63)	90 d: 27(62) / 18(63) Hospital mortality: 25(63) / 21(63)
Wang J. (2023)	Single-center, randomized controlled, open label	Esmolol; after 24 h of hemodynamic optimization, Titration to obtain HR of 80–100 bpm. Continuation until ICU discharge or severe septic shock; n = 50 (29♂/21♀)	Standard treatment; n = 50 (28♂/22♀)	16(50) / 25(50)	90 d: 29(50) / 36(50)
Cocchi (2022)	Two-center, randomized, placebo controlled open label	Esmolol; Titration to obtain HR of 80–94 bpm, n = 18 (10♂/8♀)	Placebo; n = 22 (13♂/9♀)		Hospital mortality: 6(18) / 8(22)
Kakihana (2020)	Multicenter, Randomized controlled, open label	Landiolol; within 96 h after randomization Titration until HR decreased to less than 95 bpm. After 96 h potential transition to other betablockers (orally/ intravenous), n = 76 (52♂/24♀)	Standard treatment; n = 75 (38♂/37♀)	9(75) / 15(75)	28 d: 9(75) / 15(75)
Wang Z. (2015)	Single-center, randomized, controlled, open label	Esmolol + Milrinone (50mg/kg/ 6h) for 4 d; n = 84 (19♂/11♀) reduction of HR to lower than the predefined threshold of 95 beats/min and maintenance within the target range between 75 and 94 beats/min during the first 96 h after a different intervention	Standard treatment; (20♂/10♀) Milrinone; (19♂/11♀). Additional Milrinone-with a loading + maintenance dose	ME: 12(30) M: 20(30) C: 22(30)	12(30) / 42 (60)
Morelli (2013)	Single-center, randomized controlled, open label	Esmolol; after 24 h of hemodynamic optimization; Titration to maintain HR of 80–94 bpm, continued until ICU discharge or severe septic shock; n = 77 (54♂/23♀)	Standard treatment; n = 77 (53♂/24♀)	38(77) / 62(77)	28 d: 38(77) / 62(77) Hospital mortality: 52(77) / 70(77)

bpm, beats per minute; d, day; h, hour; HR, heart rate; IQR, interquartile range; n, number of patients; ♂, male participants; ♀, female participants; C, Control; M, Milrinone; ME, Milrinone + Esmolol

Interventions were almost consistent with regard to the target heart rate which was achieved through relatively similar protocols. Most studies titrated the intravenous betablocker to obtain the target heart rates of 80–94 bpm [11, 13–17] or < 100 bpm [12]. Treatment was initiated after hemodynamic stabilization and continued for as long as vasopressors were required, up to 96 h, or until ICU discharge (Table 1). Interestingly, Wang et al. investigated milrinone as an additional study drug [12]. For the purpose of this meta-analysis, patients treated with esmolol + milrinone were compared to those receiving milrinone only. Overall, more male (61.5%) than female patients were included, and a lower average patient age was observed in three studies [11, 13, 15]. The main cause for sepsis was considered to be located pulmonary. (Table 2). Also, most studies reported no substance-related adverse events or serious adverse events [11, 15–17]. One study reported two cases of asymptomatic bradycardia [12]. Notably, Whitehouse et al. reported 25.4% of patients in the landiolol group experienced serious adverse events, compared to 6.4% in the standard group. The most frequently observed adverse event was hypotension, which was deemed to be a direct consequence of the administration of landiolol. Additionally, a potential causal relationship has been suggested between the administration of landiolol, heart failure, and myocardial infarction [14]. Finally, even though Kakihana et al. reported no significant difference in adverse events between groups, serious adverse events associated with landiolol were documented in 6% of the patients, predominantly manifested as hypotension, followed by cardiac arrest, bradycardia, and ejection fraction reduction [13]. We were able to obtain individual patient data from three studies [13–15] in order to compare the effect of the cardiac rhythm on mortality. A total of 470 patients were included in this subgroup analysis (Atrial fibrillation: 20% versus sinus tachycardia: 80%).

**Table 2:** Study characteristics, patient’s age and cause of sepsis

Author	Age	Cause of sepsis		
Rehberg (2024)	L: 64.4 ± 12.5 C: 65.2 ± 15.0.6	L: 42.9% lung 40.8% abdominal 16.3% urine 4.1% surgical 15.3% other		C: 44.9% lung 30.6% abdominal 11.2% urine 9.2% surgical 18.4% other
Whitehouse (2023)	L: 55.9 ± 16.2 C: 55.3 ± 17.1	L: 44.4% lung 33.3% abdominal 12.7% other 6.3% urine 3.2% blood		C: 42.9% lung 34.9% abdominal 20.6% other 1.6% urine
Wang J (2024)	E: 69 (IQR:58–77.25) C: 67.5 (IQR 56.7–5-77	E: 38% lung 4% hepatapostema 6% cholangitis 9% peritonitis 1% nephropyelitis 11% other		C: 48% lung 3% hepatapostema 6% cholangitis 11% peritonitis 3% nephropyelitis 3% other
Cocchi (2022)	E: 62 (IQR:53–67) C: 64 (IQR:59–71)	E: 33% lung 33% urinary 28% abdominal 17% skin 11% other		C: 32% lung 9% urinary 32% abdominal 5% skin 27% other
Kakihana (2020)	L: 67.8 ± 13.8 C: 66.4 ± 15.2	L: 29% lung 24% abdominal 12% urinary 11% skin 1% catheter-related 1% bone 13% unknown 9% other		C: 31% lung 21% abdominal 20% urinary 5% skin 3% catheter-related 1% bone 15% unknown 3% other
Wang Z (2015)	E: 34 (range; 21–60) C: 33.5 (range; 23–60) M: 38 (range; 20–57)	ME: 46.7% lung 26.7% abdominal 13.3% catheter-related 6.7% bone and joint 6.7% skin	M: 50% lung 30% abdominal 13.3% catheter-related 3.3% bone and joint 3.3% skin	C: 46.7% lung 26.7% abdominal 16.7% catheter-related 6.7% bone and joint 3.3% skin
Morelli (2013)	E: 65 (IQR:52–75) C: 69 (IQR:58–78)	E: 70.1% lung 27.3% abdominal 1.3% urinary 1.3% necrotizing fasciitis		C: 57.1% lung 39% abdominal 1.3% urinary 2.6% necrotizing fasciitis

L, Landiolol; C, Control; E, Esmolol; M, Milrinone; ME, Milrinone + Esmolol

**Fig. 2:**
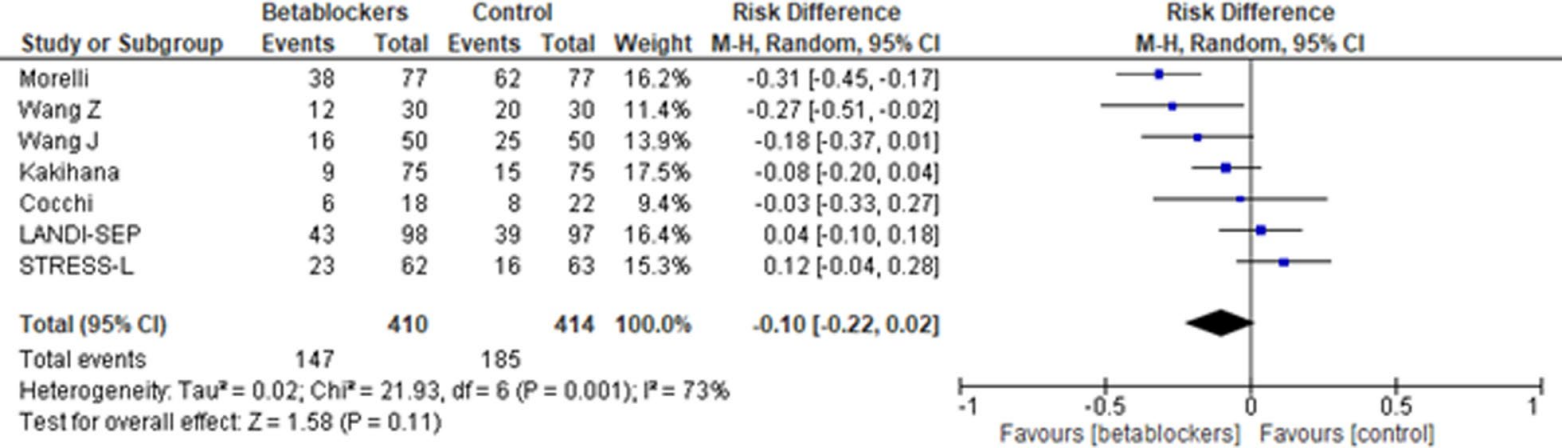
Short-term mortality. Risk difference, short-acting betablocker treatment versus Control; M–H, Mantel–Haenszel; CI, confidence interval

**Fig. 3:**
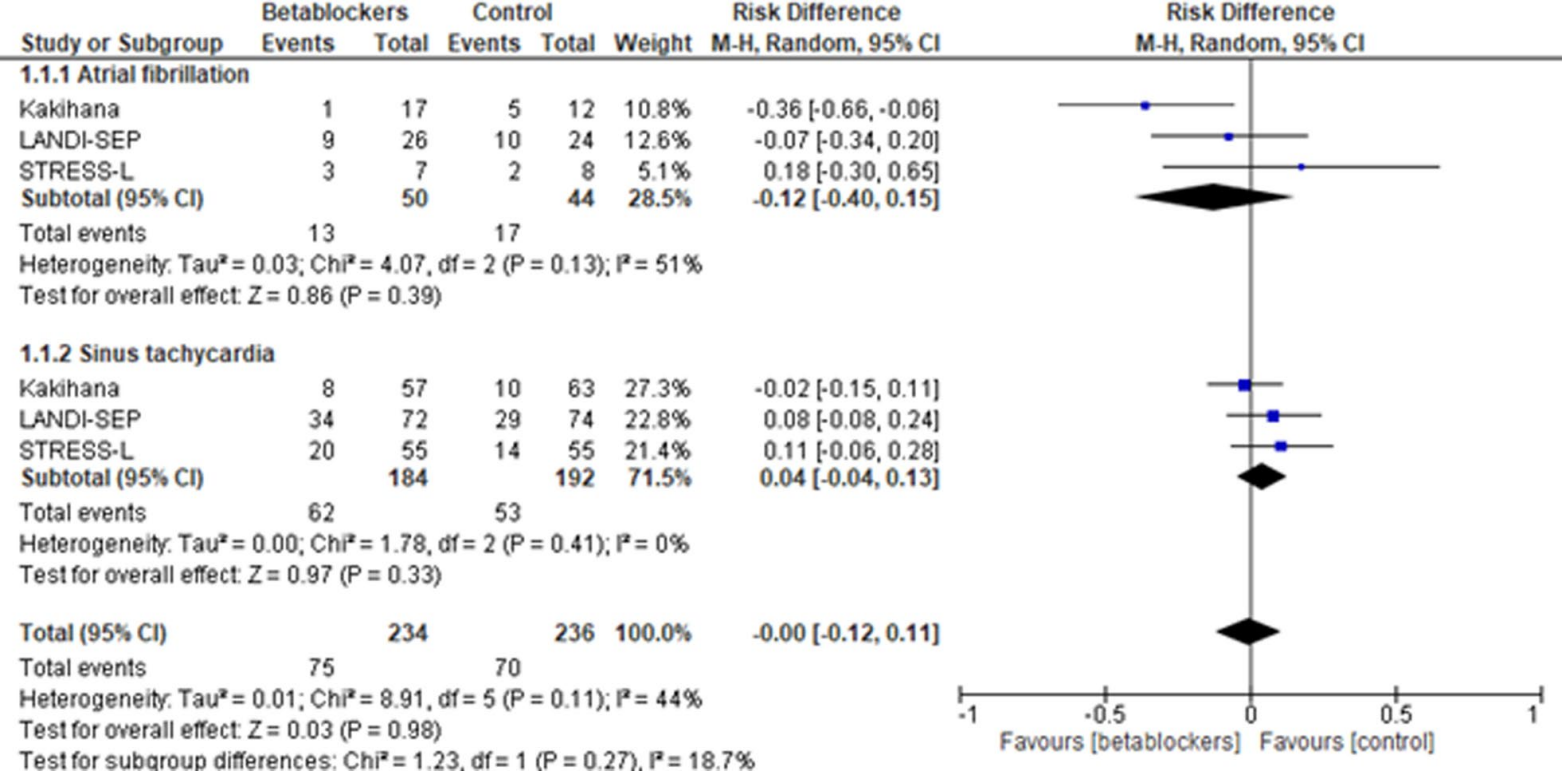
Short-term mortality. Subgroup analysis comparing atrial fibrillation with sinus tachycardia. M–H, Mantel–Haenszel; CI, confidence interval

Analysis of short-term mortality (Fig. 2) did not indicate a significant difference in patients treated with short-acting betablockers compared to standard treatment (Risk difference, − 0.10 [95% CI, − 0.22 to 0.02]; *p* = 0.11; *p* for Cochran Q = 0.001; I^2^ = 73%). No significant differences between subgroups could be observed when comparing atrial fibrillation with sinus tachycardia (*p* = 0.27; Fig. 3) and in patients < 65 versus ≥ 65 years of age (*p* = 0.19; Supplement Fig. 1).

**Fig. 4:**
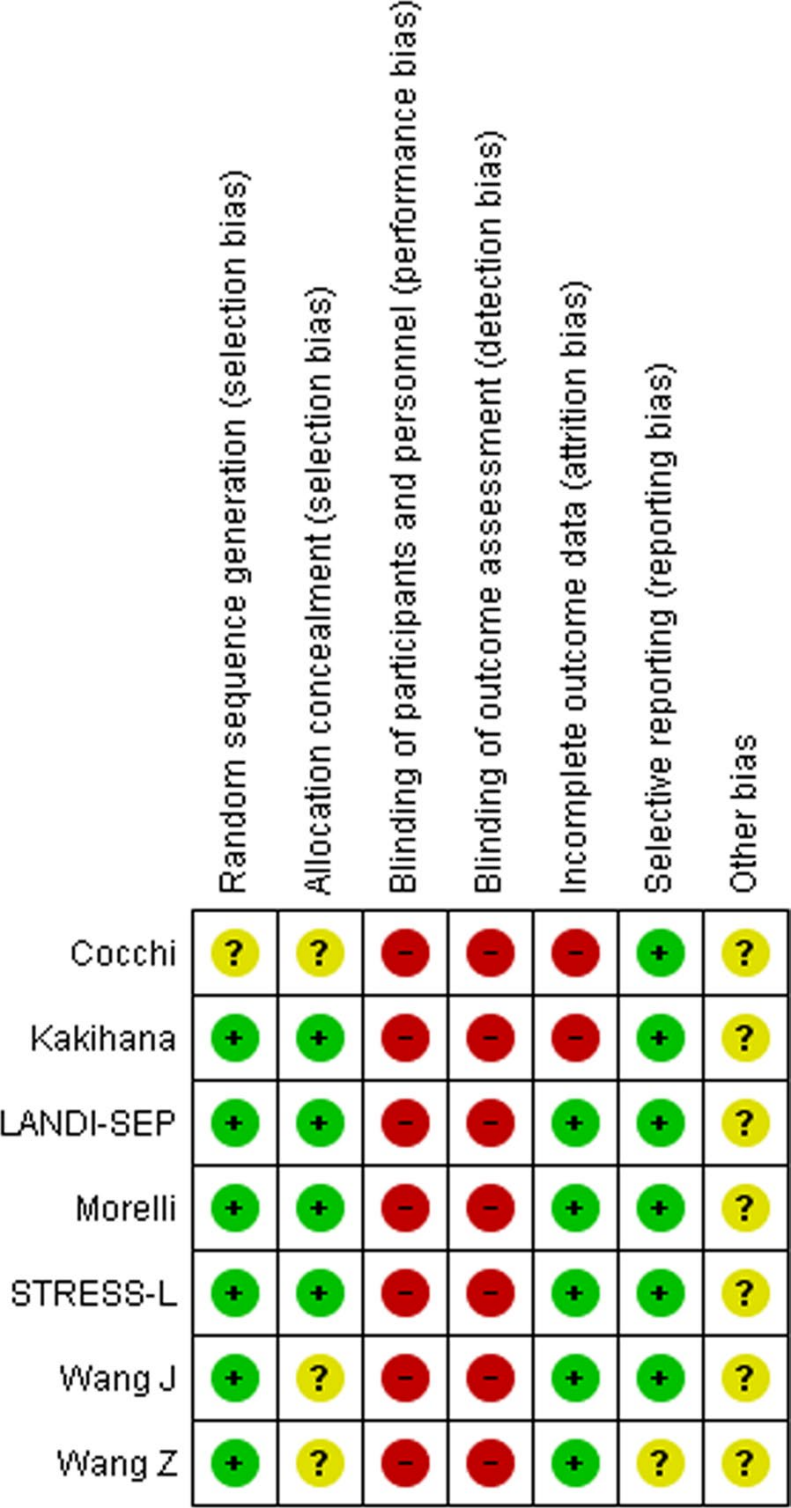
Risk of Bias assessment

Sensitivity analyses were conducted to examine the impact of the timing of mortality. These did not reveal any statistically significant differences in mortality rates between the various time periods, as indicated by the 90-day mortality rate (Supplement Fig. 2), hospital mortality rate (Supplement Fig. 2), or pooled mortality rate (Supplement Fig. 2), which represents the longest period for which mortality data were available. The overall bias was judged as moderate-to-high as all studies were performed in an open-label fashion (Fig. 4). No evidence of significant publication bias (Supplement Fig. 3) was observed.

## Discussion

The present meta-analysis summarizes randomized controlled trials published in English on the impact of short-acting betablockers on mortality in septic patients with persistent tachycardia, including the two recently published multicenter studies STRESS-L and LANDI-SEP. The results of our study suggest no reduction in short-term mortality within the included patient population. We also did not observe any statistically significant effect on hospital mortality, 90-day mortality, nor pooled mortality. Subgroup analyses did not reveal any differences in patients < 65 versus ≥ 65 years of age or if the initial cardiac rhythm was atrial fibrillation or sinus tachycardia.

Our results differ from previous meta-analyses that indicated significantly reduced overall mortality [25] or 28-day mortality [26]. These differences may be attributed to our strict inclusion criteria. As septic patients are a heterogenous population, cautious consideration of inclusion criteria is mandatory in order to achieve the highest quality of data. Therefore, we focused on randomized controlled trials and, primarily, on short-term mortality. Our results are heavily impacted by the two most recent multicenter trials [14, 15] that were not included in the former meta-analyses, and which did not reveal any mortality reduction in septic patients [25, 26]. In this context, the effects of the STRESS-L study need to be discussed in more detail, as it was stopped prematurely due to a signal of possible harm in the intervention group. Twenty-eight-day mortality rate was only 25.4% in the control group (compared to 37.1% in patients treated with landiolol), which is very low compared to other studies in septic populations. The authors acknowledged that they were unable to provide an explanation for this discrepancy. The representativeness of the control group in the STRESS-L study has been questioned [32]. In addition, no data on cardiac output was reported and individual responses to landiolol are unknown. This discussion further highlights the importance of thoroughly analyzing current evidence in order to define future treatment strategies and to identify patients who may benefit from this treatment.

Heart rate control in atrial fibrillation using betablockers is already a well-established therapeutic approach [33]. Interestingly, secondary analysis concerning cardiac rhythm at randomization indicated no significant effect on mortality, which may indicate a different response in patients with both sepsis and atrial fibrillation. However, this subgroup analysis is limited by the small sample size as well as considerable heterogeneity. The latest randomized controlled trials provided relatively homogenous inclusion criteria and study protocols, starting betablocker therapy after initial fluid resuscitation and titrating the drug to a target HR between 80 and 94 bpm. Interestingly, at least two of the Esmolol studies used levosimendan or milrinone [11, 12]. Hence, one may hypothesize that concomitant use of inotropes may be beneficial. Unfortunately, further data on this issue is lacking. While some trials delivered promising results while treating patients with esmolol [11, 12, 16], the largest multi-centered trials did not report a significant mortality reduction in the treated populations using landiolol [13–15]. This is further supported by the results of our primary analysis. Based on these observations, two possible hypotheses could be postulated. First, esmolol may be more beneficial than landiolol, although the pharmacological properties of landiolol demonstrate superior selectivity, potency, and additional beneficial pharmacokinetic effects when compared to esmolol [34, 35]. However, one could hypothesize that less beta_1_-selectivity could also be beneficial. Notably, 25.4% of the patients treated with landiolol in the LANDI-SEP study presented serious drug-related adverse events, including hypotension and bradycardia. Secondly, the discrepancy between the results could be a problem of correct patient identification. Monitoring stroke volume would indicate any significant benefit or compromise from heart rate reduction as the tachycardia may be due either to sympathetic stimulation or compensatory to a sepsis-induced cardiomyopathy [36] impaired cardiac function. The use of left ventricular ejection fraction (LVEF) in this population is currently under investigation in the ongoing HyperBetashock trial (NCT04748796) although LVEF has its own limitations due to reduced afterload attributed to the distributive shock in sepsis [36]. One might suggest that other echocardiography measurements of diastolic dysfunction, such as septal relaxation e’ or speckle tracking, which remain afterload-independent, may be superior [36]. Of the seven studies included in our meta-analysis, only three provided data on echocardiography [13, 15, 16]. Of these, only Wang J. et al. used additional diagnostic methods. Using the proper tools to assess cardiac dysfunction may play a pivotal role in selecting patients who may benefit from beta_1_-blockade. Based on current data, however, these hypotheses remain purely speculative but are worth exploring in future investigations. Furthermore, the potential correlation between the efficacy of heart rate reduction and catecholamine usage or mortality remains a topic of particular interest. However, due to the incomplete availability of individual data, this could not be addressed in the present analysis.

The present results are limited by the open label design of the included randomized controlled studies. The secondary analysis regarding patient age is limited due to missing individual patient data. Hence, we categorized patients based on all available data in order to provide a sensitivity analysis on potential biological heterogeneity. As with all meta-analyses, the risk of publication bias has to be considered, however no significant publication bias was determined. A key limiting factor when analyzing mortality rates in sepsis is the inherent heterogeneity, as previously discussed.

## Conclusions

In this meta-analysis, heart rate control with short-acting betablockers did not reduce mortality in septic patients. Underlying mechanisms should be further evaluated in future studies. These need to provide extensive data on hemodynamic monitoring, cardiac function, and individual patient data to support an individualized approach in order to identify patients that may benefit from this therapeutic regimen.

## Supplementary Information

Additional file1.

## Data Availability

No datasets were generated or analysed during the current study.

## Abbreviations

SD: Standard deviation
bpm: Beats per minute
MAP: Mean arterial pressure
HR: Heart rate
SIRS: Systemic inflammatory response syndrome
LVEF: Left ventricular ejection fraction
