# Supplementary material for: Mortality in septic patients treated with short-acting betablockers: a comprehensive meta-analysis of randomized controlled trials
Source: Crit Care. 2024 Nov 27;28:392. doi: 10.1186/s13054-024-05174-w (PMC11603935; doi:10.1186/s13054-024-05174-w)
Supplement: Supplementary file 1 — Additional file1. [file 13054_2024_5174_MOESM1_ESM.docx]

**Supplement:**


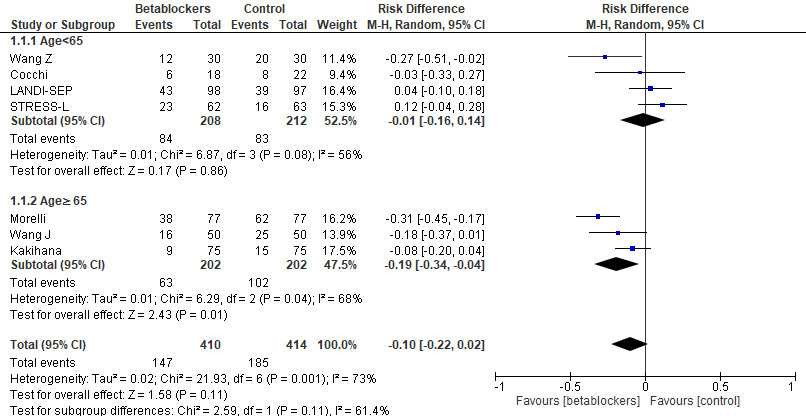


Supplement Fig. 1 Short term mortality. Sensitivity analysis regarding mean patient age of the included studies; Risk difference, betablocker treatment versus Control; M-H: Mantel-Haenszel, CI: confidence interval.


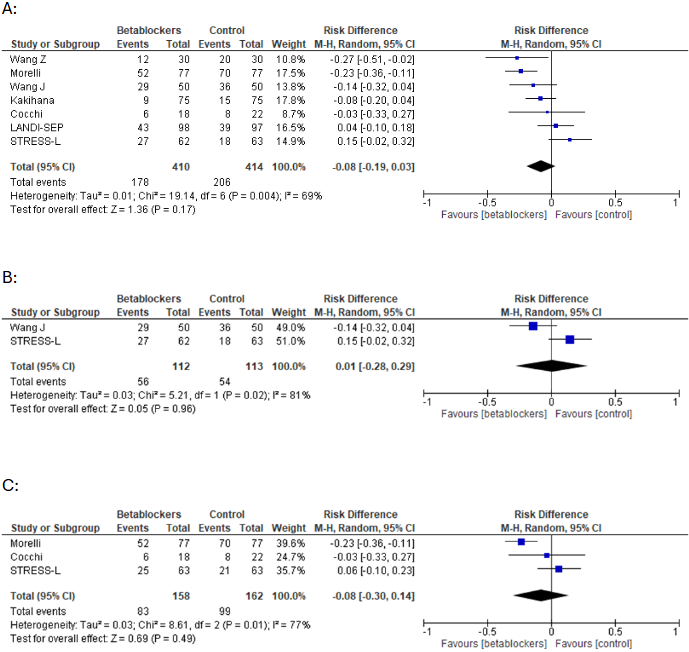


Supplement Fig. 2 Sensitivity analyses. A: Pooled mortality (longest period of data on mortality); B: 90-day mortality; C: Hospital mortality; Risk difference, betablocker treatment versus Control; M-H: Mantel-Haenszel, CI: confidence interval.


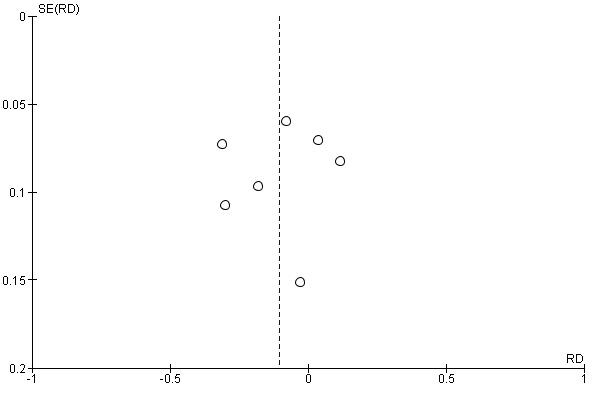


Supplement Fig. 3 Funnel plot.

Supplement table 1. Study characteristics, inclusion and exclusion criteria

| Author | Inclusion criteria | Exclusion criteria |
| --- | --- | --- |
| Rehberg  (2024) | Age ≥18 years, septic shock, acute increase of ≥2 points on SOFA; Need for vasopressor therapy for MAP of >65 mmHg despite adequate fluid resuscitation; Blood lactate >2mmol/L Tachycardia and/or tachyarrhythmia with heart rate ≥95 bpm, NA infusion. Hemodynamic optimization period of at least 12 hours (maximum of 36 hours) | Compensatory tachycardia, betablocker treatment after septic shock diagnosis, Sick Sinus syndrome, severe atrioventricular disorders (without pacemaker). A known serious cardiovascular condition such as ischemic stroke or transient ischemic attack within the last 6 months, or pre-existing heart failure NYHA IV, Cardiogenic shock |
| Whitehouse  (2023) | Aged 18 years or above STRESS-L Protocol; Being treated on an ICU; Septic shock according to internationally accepted definitions (Sepsis-3 definition); Heart rate ≥95 bpm (at the time of randomization); Receiving vasopressor support to maintain a target blood pressure for ≥24 hours; Are being treated with noradrenaline at a rate ≥ 0.1mcg/kg/min | Tachycardia as a result of pain, discomfort from medical devices (including endotracheal tubes), during interventions or other patient distress; Any form of vasodilatory shock that is not caused by sepsis; Noradrenaline infusion <0.1mcg/kg/min x >72 hours after start of vasopressor therapy; <12 hours since noradrenaline to treat a medical condition after than septic shock stopped; Having pre-existing severe cardiac dysfunction (NYHA grade 4 or more); Having pre-existing severe pulmonary hypertension (mean PA pressures > 55mmHg); Acute severe bronchospasm (due to asthma or COPD); Untreated second or third degree heart block; Untreated phaeochromocytoma; Prinzmetal's angina; A past history of ischaemic stroke or transient ischaemic attack (TIA) or untreated severe carotid stenosis.; Advanced liver disease with Child-Pugh Score of ≥B; Known sensitivity to beta-blockers; Patient / legal representative unwilling to provide written informed consent; Known to be pregnant; Terminal illness other than septic shock with a life expectancy < 28 days; Participants who have been administered an investigational medicinal product for another research trial in the past 30 days; Patients in whom the clinical team feel are about to finish their noradrenaline therapy; Decision of withdrawal of care is in place or imminently anticipated; Receiving extracorporeal membrane oxygenation (ECMO) treatment |
| Wang J  (2023) | Age ≥18 years, Sepsis/ Septic shock based on Sepsis-3, within 1h after admission + Tachyarrhythmia HR ≥ 100 bpm, after completing 1h bundle and 24h of standardized treatment according to Surviving Sepsis Campaign 2018. LVEF ≤45% or GLS≥-19% | History of AF, long term betablockers, preexisting heart failure NYHA ≥ III, valvular heart disease; rapid arrhythmias such as new-onset atrial fibrillation/atrial flutter; new-onset cardiogenic shock (LVEF ≤25%); poor echocardiography image quality; refusing to sign informed consent; treatment in other hospital > 48h |
| Cocchi  (2022) | Age ≥18 years, Sepsis; Need for vasopressor therapy for MAP of >65 mmHg despite (minimum 0.1 mcg/kg/min NA) and 65 mm Hg despite adequate fluid resuscitation; Tachycardia HR ≥95 bpm. If patients were on additional vasopressors, those doses were converted to norepinephrine equivalent doses | Betablockers prior to randomization, pre-existing heart failure, valvular heart disease, known allergy to esmolol, asthma, COPD, DNR or DNI, were receiving an infusion of epinephrine, dopamine, dobutamine, or milrinone at time of enrollment, clinical team refused treatment (goals of care) or the patient was moribund on maximal vasopressor therapy |
| Kakihana  (2020) | Age >20, Sepsis, acute increase of ≥2 points on SOFA; Need for vasopressor therapy for MAP of >65 mmHg, Tachycardia ≥100 bpm + Confirmation of the mentioned symptoms and signs within 24 h before randomization and within 72 h after entering an ICU. | Patients with bradyarrhythmia, long term betablockers; Sick Sinus syndrome, pacemakers and/ or cardiac defibrillators; class IV antiarrhythmics, digitalis were prohibited up to 96h after randomization except for treatment of adverse events. |
| Wang Z  (2015) | Age ≥18 years, Sepsis; Tachycardia ≥90 bpm/ ≥95 bpm after early goal-directed therapy | Sick Sinus syndrome, severe atrioventricular disorders, asthma history, a history of allergic reaction to milrinone or esmolol |
| Morelli  (2013) | Age ≥18 years, Sepsis; Need for vasopressor therapy for MAP of >65 mmHg despite adequate fluid resuscitation; Tachyarrhythmia HR ≥ 95 bpm. | Betablockers prior to randomization, pre-existing heart failure, valvular heart disease and pregnancy. |

Abd: Abdominal; AF: atrial fibrillation; BB: betablockers; COPD: chronic obstructive pulmonary disease; DNI: do-not-intubate; DNR: Do-not-resuscitate; HR: heart rate; ICU: intensive care unit; LVEF: left ventricle ejection fraction; MAP: mean arterial pressure; NA: norepinephrine; NYHA: New York Heart Association; SOFA: Sequential Organ Failure Assessment.
